# An Interpersonal CBT Framework for Involving Relatives in Interventions for Psychosis: Evidence Base and Clinical Implications

**DOI:** 10.1007/s10608-015-9731-3

**Published:** 2015-12-11

**Authors:** Fiona Lobban, Christine Barrowclough

**Affiliations:** Division of Health Research, Faculty of Health and Medicine, Spectrum Centre for Mental Health Research, Lancaster University, Lancaster, LA14YT UK; School of Psychological Sciences, University of Manchester, Manchester, UK

**Keywords:** Psychosis, Relatives, Recovery, CBT, Family intervention, Interpersonal

## Abstract

Working with families in psychosis improves outcomes and is cost effective. However, implementation is poor, partly due to lack of a clear theoretical framework. This paper presents an interpersonal framework for extending the more familiar cognitive behavioral therapy model of psychosis to include the role of relatives’ behavior in the process of recovery. A summary of the framework is presented, and the evidence to support each link is reviewed in detail. Limitations of the framework are discussed and further research opportunities highlighted. Clinical implications and a case example are described to show how the framework can be used flexibly to facilitate clinical practice. Our aim is to shift the focus of psychosocial interventions from an individualistic approach to treatment, towards greater involvement of relatives and recognition of the importance of the social environment on mental health.

**Electronic supplementary material:**

The online version of this article (doi:10.1007/s10608-015-9731-3) contains supplementary material, which is available to authorized users.

## Introduction

Both cognitive behavior therapy (CBT) and structured family interventions (FI) are recommended psychological treatments for psychosis in clinical guidelines around the world (Gaebel et al. 2005). However, equally widespread is evidence of poor levels of implementation of both CBT and FI (Drake et al. 2009; Kuipers 2011; Mojtabai et al. 2009; Resnick et al. 2005) and recognition of the urgent need to find ways to increase availability (The Schizophrenia Commission 2012; Dausch et al. 2012; Farhall and Thomas 2013).

Of these two recommended interventions, there is some evidence that individual CBT has been the more successfully implemented, particularly in the UK National Health Service (NHS) (Haddock et al. 2014). It benefits from: a strong and extensive evidence base for effectiveness in symptom reduction when compared to both treatment as usual (TAU) (Wykes et al. 2008), and some evidence of superiority over other psychological interventions (Hutton 2013; Jauhar et al. 2014; Jones et al. 2012); an underlying theoretical framework consistent with CBT models for a wide range of other mental health problems for which CBT treatments have also been shown to be effective and are widely used; and clear intervention strategies which target specific measurable outcomes that can be clearly defined for health services driven by the need to provide quantifiable evidence of effectiveness.

FI have fared less well. Despite an equally strong evidence base for both clinical and cost effectiveness (Andrew et al. 2012; Pfammatter et al. 2006; Pharoah et al. 2010), local initiatives to improve access (Allen et al. 2013; Dixon et al. 2014; Fadden and Heelis 2011), and accessible self-management toolkits (Lobban et al. 2013a), implementation levels internationally are very poor. This problem needs addressing because as well as clear benefits for service users, FI also improves important outcomes for family members (Addington et al. 2005; Lobban et al. 2013b), who continue to provide the vast majority of care for people with mental health problems worldwide.

There are a number of reasons why FI are more difficult to implement than individual CBT including: individualistic models of care in health services in which clinician caseloads are measured in terms of individual service users seen, not accounting for family members; lack of training for staff resulting in low confidence; and fear in service users and family members about what the process will involve leading to a reluctance to engage (Fadden 2006; Glynn et al. 2006). However, we believe that another key barrier is the lack of familiarity with the theoretical model underlying FI among psychological therapists, who consequently feel less confident in using this approach in routine clinical practice. Without a clear framework, the involvement of family members in therapy can feel unstructured, unpredictable, and challenging. Psychosis is not straightforward to describe, diagnose, explain or treat, and consequently relatives may be distressed, frustrated, even angry with services, and may be assertive in seeking definitive answers which clinicians are unable to provide.

### Aim of Paper

To address this barrier, we present a framework for extending the CBT model to include the role of relatives’ behavior in the process of recovery in psychosis. Whilst most CBT models do highlight the very significant role of the social environment as an important determinant of an individual’s thoughts and behaviors, both concurrently and prospectively, the focus of the intervention is primarily (though not exclusively) on changing the individuals’ thoughts and behaviors directly, rather than on modifying the social environment. Our interpersonal framework highlights the additional opportunities for intervention offered by this more systemic approach. Our aim is to improve outcomes for both people with mental health problems and relatives by shifting the focus in psychosocial interventions from an individualistic approach to treatment, to one that has a greater focus on the importance of the social environment and which encourages more involvement of relatives.

A summary of the framework is presented, and the evidence to support each hypothesised link (numbered to aid cross referencing between the figure and the text) is reviewed in detail. We do not present any new primary data, and the framework is likely to be familiar to clinicians as it has been presented previously in conference workshops (Lobban 2012; Lobban and Barrowclough 2007, 2008–2009), draws on previous systemic frameworks (Barrowclough and Tarrier 1992), and has been cited as informing the development of other models, such as the Cognitive Interaction Model (Burbach 2012, 2013). However, this is the first attempt to synthesise existing evidence to test the validity of this framework, and to highlight opportunities for further research which will help to progress an evidence based approach towards working with relatives which is rooted in a theoretical framework. Finally, we describe clinical implications and a case example to show how the framework can be used flexibly to facilitate clinical practice.

### Definition of Terms

We use the term “service user” to refer to a person with a mental health problem who is seeking help. We have focused primarily on service users with psychosis in order to build a coherent argument and because it is the literature with which we are most familiar. However, we believe that the framework outlined is equally valid across all diagnostic groups, though the specific content of the beliefs, behaviors and emotions will differ. We use the term “relative” to refer to any person with a close relationship to the service user and who plays a direct role in supporting them. This person may not be directly related by genes or marriage, and could include a close friend or partner.

### Summary of an Interpersonal Cognitive Behavioural Framework

Many CBT models to explain the maintenance of psychosis, have been proposed, most notably those by Morrison (2001), Garety et al. (2001), Freeman et al. (2002), Steel et al. (2005), and Bentall et al. (2001). These excellent reviews present the evidence to support an individual CBT model for psychosis in detail and consequently, this will not be repeated here. In summary, a trigger (internal, such as a normal intrusive thought or image, or external, such as the behavior of another), is misinterpreted by the service user. This appraisal is driven by information processing biases, which are themselves influenced by the social environment. Social isolation and interpersonal experiences throughout life which inform the development of schematic beliefs about self, others and the world, are hypothesised to increase vulnerability to misinterpretation occurring. The misinterpreted event generates negative emotional responses, often distress or fear, which in turn drive behaviors in an attempt to cope. Often these attempts to cope serve only to reinforce the misinterpretation and maintain distress. For example, intrusive thoughts about being a bad person may be experienced as not being generated by the self and experienced instead as an external voice. Attempts to cope may include social withdrawal or shouting back and arguing against the voice. If the voice makes commands, this can lead to bizarre and even risky behavior if the person feels they must respond to command hallucinations. A similar process is hypothesised to underlie the maintenance of delusional beliefs. In this case, an external event, such as the behavior of another person is misinterpreted, driving the emotional and behavioral responses. For example, a benign approach from a stranger or a kind gesture from a relative may be misinterpreted as a threatening intrusion, and lead to fear and withdrawal or even aggression. Clinical interventions can focus on challenging the interpretations of the trigger events, modifying the underlying schematic beliefs thought to drive the misinterpretations, and developing alternative behavioral responses to cope with the experience.

**Fig. 1:**
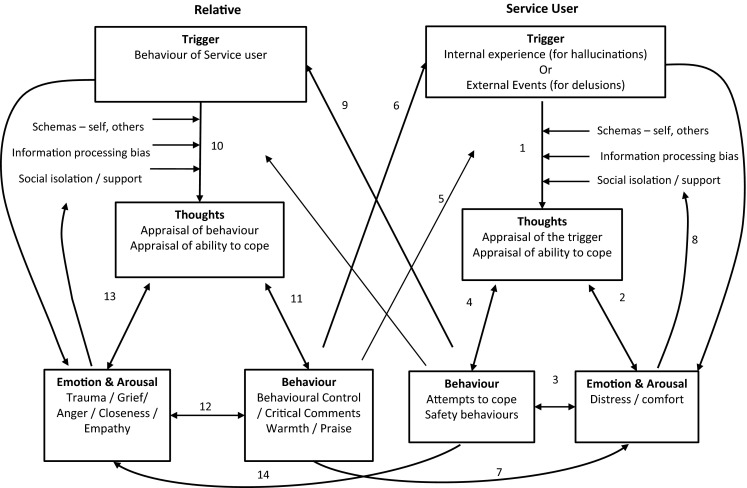
Interpersonal CBT framework. *Numbered arrows* are referred to in the text to link the figure with evidence for *each arrow*

Figure 1 provides a summary of the interpersonal CBT framework that will be developed in subsequent sections of this paper. It retains the key elements of the individual CBT model outlined above, but elaborates the model to include parallel psychological processes for the relative, and the consequent dynamic interactions between relative and service user. In summary, we propose that relative’s behavior impacts on service users in three ways. Firstly, relatives’ general patterns of behavior can influence the content of schemas and processing of information (link 5). Where service users are surrounded by affection and positive feedback, they are likely to see themselves as loveable and others as safe and a source of comfort. The presence of relatives can reduce social isolation and they can offer benign alternative appraisals of ambiguous events, preventing psychotic misinterpretations. In contrast where relatives are somewhat critical, this will generate negative schemas about self and others which may contribute to or reinforce service user negative appraisals of triggers. Repeatedly negative behaviors from relatives may be generalised by service users and serve to reinforce negative interpretation of benign events, driving psychotic paranoia. Secondly, specific behaviors from relatives may act as the direct triggering event for a psychotic experience (link 6). Finally, relatives’ behavior could increase negative emotion and arousal levels in the service user (link 7), which in turn impacts on information processing skills (link 8).

Our framework highlights the dynamic interaction between service users and relatives’ behavior. There is evidence that certain service user behaviors are more likely to elicit negative responses from relatives than others (link 9). Consistent with the cognitive model, however, it is the relatives’ appraisals of these behaviors as being controllable and the personal responsibility of the service user (link 10) that seem to drive the negative behavioral responses (link 11) in the relative. These behaviors are also thought to be attempts by relatives to manage the intense emotional responses to psychosis including anxiety, fear and grief (link 12). Conversely “survivor appraisals”, in which service users are viewed as responsible for positive events but not for negative events, are more closely associated with warmth and positive feedback from relatives.

Consistent with more systemic models of human cognition and affect, such as the Interacting Cognitive Subsystems (ICS) (Barnard and Teasdale 1991; Gumley et al. 1999) and the Schematic, Propositional, Analogical, and Associative Representation Systems (SPAARS) model (Power and Dalgleish 1999), Fig. 1 also includes links to account for direct emotional responses that relatives may have to service users’ behavior (link 14), and vice versa (link 7), that occur alongside those mediated by appraisals. Our review focuses on evidence that is relevant to the interpersonal dimension of the framework presented in Fig. 1. Evidence for each of the numbered links will be reviewed below.

We have deliberately chosen to focus only on the impact of ongoing relationships and the role they play in current distress. We do not review the extensive literature on the impact of early relationships on psychosis, or propose a mechanism for the development of psychosis linked to early relationships. Historical relationships, whilst undoubtedly highly significant, are not amenable to direct change. Understanding service users’ current distress as a function of past events may provide insight and self-compassion for some people, and where there has been significant trauma or abuse, this may be an essential part of recovery. However, for many others, focussing primarily on understanding current mental health as a function of early experiences can cause anger and blame, which can further destroy rather than build potential support networks especially within families.

Where possible we try to highlight evidence to demonstrate the beneficial impact of supportive interpersonal relationships. Specifically we explore the impact of positive relationships on the development of schemas, and the role of supportive relatives’ behaviors in triggering virtuous cycles that may facilitate wellbeing and resilience. We do this in order to encourage the use of positive formulation in working with relatives in which examples of successes can be explored alongside examples of problems. We also extend the concept of recovery to include the relatives, and consider the impact of any interpersonal processes on their wellbeing too. This more solution focussed approach can facilitate engagement with relatives who often fear they will be blamed for the service users mental health difficulties and can facilitate change more effectively.

## Evidence to Support an Interpersonal CBT Framework

### Relatives’ Current Behavior Impacts on the Service User

Relationships with family, friends and peers all play a significant role in psychosis. Firstly the mere presence of close relationships and friends seems to be important. For example, there is evidence that social isolation, particularly in minority immigrant populations is associated with increased risk of psychosis (Cantor-Graae and Selten 2005), whereas living with a relative is associated with significantly better outcome. Social support from family and close friends during the early stages of psychosis predicts better functioning 5 years later, even controlling for other variables such as education, duration of untreated psychosis, symptoms and baseline functioning (Norman et al. 2012). Using more real world momentary methods of assessment, being in the presence of familiar people, rather than alone or with strangers decreases risk of experiencing delusions in people with chronic psychosis (Myin-Germeys et al. 2001), and in those at risk of psychosis, the presence of familiar friends or family reduces paranoid thinking (Collip et al. 2011) and reporting of unusual experiences (Verdoux et al. 2003). A large multisite RCT testing the effectiveness of CBT and FI for people who had recently relapsed with non-affective psychosis, found no effect of either treatment on outcome, but people with an identified close relative had a significantly better outcome than those without, and the presence of a relative was associated with a more positive response to either treatment (Garety et al. 2008). There are many potential confounds that could account for these findings, but the positive impact of social support is fairly robust.

A number of theories have been put forward as to how relatives’ support may improve outcome. Unsurprisingly, where relatives are present, the quality of relationship is crucial, and most research in this area has focussed on the concept of Expressed Emotion (EE). EE is a measure of the emotional response of relatives towards the service user, rated from relatives’ reports during the Camberwell Family Interview (CFI; Leff and Vaughn 1985; Vaughn and Leff 1976). Relatives’ are rated along five scales: hostility, criticism, over-involvement, warmth and positive remarks, and those who score six or more on critical comments, any hostility, or a rating of three or more on emotional over involvement (EOI), based on overprotective, excessively devoted or self-sacrificing style towards the service user are described as high EE, compared to low EE relatives who do not meet this criteria. Interestingly, ratings of warmth or positive remarks do not contribute to the EE rating. Early studies in the 1960s first measured the importance of the family environment for people with schizophrenia (Brown and Rutter 1966) and a meta-analysis of 26 studies in this area concluded that living in a high EE critical or hostile home environment more than doubles the risk of relapse over 9–12 months for people with psychosis (Butzlaff and Hooley 1998). Further, interventions that reduce high EE can significantly improve outcome for service users (Hooley 2007), supporting a causal role for relationship quality in relapse.

The exact mechanism by which EE predicts relapse is not yet clear. Attempts have been made to observe differences in behavior towards the service user between high and low EE relatives to see if specific behaviors can be identified that could play a role in the relapse process and which could be targeted in interventions with relatives. Using methods of coding relatives’ behavior during an interaction with the service user, such as the Kategoriensystem für Parnerschaftliche Interaktion (KPI; Interaction Coding System; Hahlweg and Conrad 1985), relatives categorised as high EE on the CFI, or rated as having a higher level of criticism considered alone, demonstrate higher levels of negative verbal or nonverbal behavior when compared with low EE or less critical relatives (Hahlweg et al. 1989; Hooley 1986; Mueser et al. 1993; Simoneau et al. 1998). Using an alternative rating of behavioral control based on coding statements from the CFI interviews, Hooley and Campbell (2002) found that high EE relatives behaved in a more controlling manner than low EE relatives. Furthermore, behavioral control was a significant predictor of relapse at 9 months. The association between high EE relatives and the use of more controlling behaviors has been replicated in a sample of people with recent onset psychosis, and further developed by distinguishing behavioral styles between high EE-critical relatives and high-EE over-involved (Vasconcelos e Sa et al. 2013). Critical relatives tended to describe using more “direct influencing” in which they attempt to change the service users’ behavior using mild behaviors such as a polite request, or gentle reminder, through to extreme behaviors such as intimidation or ultimatums. Alternatively, relatives rated as high-EE–EOI used more “buffering” ways to take control, or do things for the service user, ranging from mild supervising or joint planning, to more intrusive actions like taking control of finances, or dealing with personal mail. Despite not finding a direct relationship between behavior and relapse in this sample, and a number of methodological limitations (including rating behavior and EE from the same interview transcripts), this study does support the idea that there are direct behaviors associated with EE.

Several potential processes have been suggested to explain how the relatives’ behavioural style impacts on psychosis in the service user (Garety et al. 2001). Firstly, relatives’ behavior could act to reinforce negative core beliefs about self, world and others that in turn impacts on information processing biases (link 5). For example, relatives behaving in a very critical way could reinforce beliefs about being useless or unlovable, and that others are critical or dangerous, leading to a bias towards negative interpretations of the behavior of others, and behavioral responses of withdrawal and avoidance that are likely to follow from this. There is some evidence to support this. In a cross sectional model, Barrowclough et al. (2003) showed the positive association between criticism from relatives and scores on the Positive and Negative Symptom Scale (PANSS; Kay et al. 1987) positive symptom scale was mediated by negative self-evaluation (interview based assessment). In a 5 year follow-up of the same sample, negative self-evaluation also predicted time to relapse even when controlling for baseline symptoms and duration of illness (Holding et al. 2013). Secondly, relatives’ behavior could act as a direct triggering event (link 6) which is then misinterpreted within a delusional framework. Once such a framework has been established, then even benign behaviors from relatives can be misinterpreted as malevolent, fuelling psychotic symptoms. Thirdly, relatives’ behavior could impact on psychosis by increasing negative emotion and arousal levels in the service user (link 7), which in turn impacts on information processing, including reasoning skills, generating further misinterpretation of triggering events (link 8). Brown et al. (1972) suggested that in high EE families, the environment was too over-stimulating, and that, consistent with the stress vulnerability model of psychosis (Zubin and Spring 1977), this acted as a direct trigger for psychosis. Support for this hypothesis comes from psychophysiological studies that show elevated autonomic arousal levels in service users with high EE relatives, compared to those with low EE relatives (Tarrier and Turpin 1992), and from self-reported elevated stress levels from service users in the presence of high EE relatives, compared to those with low EE relatives (Cutting et al. 2006). Further support comes from studies using functional magnetic resonance imaging (fMRI) which show enhanced activation of brain regions concerned with processing of aversive social information in response to hearing relatives’ critical comments compared to neutral comments, suggesting a potential neural basis to the impact of high EE environments on outcome (Rylands et al. 2011).

A major limitation of the EE research is that it has tended to study a dichotomy of high versus low EE focusing heavily on the characteristics of high EE including criticism, hostility and emotional over-involvement, with far less investigation of the characteristics and impact of low EE relatives, or the specific impact of warmth and positive remarks. What little has been done, has shown that this is potentially a very important area of investigation that could provide valuable insights into how to develop more effective solution focused treatments for psychosis that involve relatives. Cross sectional associations between warmth in relatives and satisfaction with life in people with psychosis have been shown (Greenberg et al. 2006), but more significantly, prospective studies show a predictive relationship which strengthens the argument for a causal link between positive family environments and outcome (López et al. 2004). In an attempt to replicate the original EE studies of Brown and Birley (1968), the link between high EE and subsequent relapse was reproduced (Bertrando et al. 1992), but in addition, the authors found that high levels of warmth reduced the risk of relapse over 9 months, and led to lower admission rates, even within families that were also rated as high EE. The protective impact of positive family environments has also been demonstrated in adolescents at risk of psychosis (O’Brien et al. 2006; Schlosser et al. 2010) and following first episode of psychosis (Lee et al. 2013), and highlighted as an important moderator of the negative impact of EOI in some cultures (Singh et al. 2013). These studies support the independence of the negative and positive ratings within EE and suggest that the tendency to categorise relatives as high or low EE is too simplistic to capture the multidimensional complexity of family relationships and how they impact on outcome.

From a more positive perspective, supportive behaviors from the relative could reduce vulnerability to psychosis by firstly challenging negative core beliefs about self/world/others and confirming more positive beliefs. Warmth and supportive behavior, would lend support to positive beliefs about the self and others and increase drive towards positive social interactions (link 5). Positive self-evaluation is an even stronger predictor of time to relapse than negative self-evaluation (Holding et al. 2013) so relatives who can build this, even in the presence of continuing negative self-evaluation may be able to increase resilience. Secondly, relatives can provide an alternative perspective to the misinterpretation of triggering events. Several groups have pointed out that social isolation reduces access to alternative and normalising explanations for anomalous experiences, and that the failure to be part of a normalising social network is one factor distinguishing those who develop psychosis from those who do not (Hodges et al. 1999; Van Os et al. 2000).

Relatives’ behavior, if supportive and calming could reduce arousal levels, increasing information processing capacity, and directly trigger positive emotions (link 7). These emotions may in turn initiate “upward spirals” of positive affect which have several potential beneficial effects (Garland et al. 2010). Firstly, the immediate cognitive and emotional benefits of positive emotion are likely to directly impact on common experiences associated with psychosis. The misinterpretation of ambiguous information has been identified as an important underlying cause of both hallucinations, in the form of misinterpretation of anomalous experiences (Morrison 2001) and delusions, in the form of cognitive biases towards jumping to conclusions (Garety et al. 2001). The broaden and build theory of positive emotion (Fredrickson 1998, 2001, 2003) postulates that, in the same way that negative emotion has been shown to narrow cognition and focus behavior to specific survival responses, positive emotion leads to a broadening of cognition and an increase in behavioral flexibility (link 8). Effects include broadening the scope of visual attention (Fredrickson and Branigan 2005; Rowe et al. 2007), expanding people’s repertoires of desired actions (Fredrickson and Branigan 2005), and their openness to new experiences (Kahn and Isen 1993), and critical feedback (Raghunathan and Trope 2002). Further effects at the interpersonal level, include an increase in people’s sense of “oneness” with close others (Waugh and Fredrickson 2006), and their trust in acquaintances (Dunn and Schweitzer 2005). Linking this to psychosis, we can see how positive emotions triggered by warm supportive relative’s behavior could reduce cognitive biases by triggering this more broaden and build perspective and consequently reduce vulnerability to psychotic experiences (link 8). A further common, and often equally debilitating experience in psychosis, is the loss of anticipatory pleasure for activities of life (Kring 1999), leading to lack of motivation to engage and general withdrawal, often referred to as negative symptoms. It is easy to see how a vicious cycle is created in which the loss of anticipatory reward and greater social withdrawal become entwined. Attempts to break this cycle often involve exposure to situations which may trigger positive affect to a level that can ignite anticipatory pleasure in future exposure (Tarrier 2010). Relatives who behave in warm supportive ways with a degree of consistency which can ignite anticipatory reward may therefore generate both immediate positive affect which in turn also increases the likelihood that the person with psychosis will expose themselves to other potentially rewarding social interactions (link 3), reducing the risk of withdrawal and isolation associated with long term mental health problems and breaking the vicious cycle thought to underlie negative symptoms.

### Impact of Relatives’ Behavior on Service User is Mediated by Thoughts

There is some evidence to suggest that the service users’ *perception* of relatives’ behavior may be more important than any objective rating of the actual behavior (link 1). In several studies exploring links between relatives’ behavior and service user outcome, where the service users’ perception of the relatives’ behavior has also been assessed, this has been a better predictor of outcome than the actual behavior. For example in the study by Barrowclough et al. (2003) examining the role of self-esteem in psychosis, relatives’ criticism (assessed using the CFI) was no longer predictive of negative evaluation of self when the service users’ *perceived* negative evaluation from the relative (based on service user interview ratings) was included into the model. Similarly, in studies by Schlosser et al. (2010) and Lee et al. (2013), it is the service users’ *perception of* criticism from the relative in at risk populations and *perceived* positive affect from relatives in early psychosis (respectively) which better predict subsequent outcome, rather than interview ratings of relationship quality. These findings could reflect a difference in relatives’ actual behavior in situ, compared to that assessed by the CFI used to rate EE. To truly test the relative contribution of actual behavior and perceived behavior in determining service user outcome, would require the experimental control of one of these variables. Clearly, impossible to do in real world settings, this has been done in virtual reality settings. Freeman and colleagues have developed a paradigm in which participants are asked to judge and respond to the behavior of avatars in computer generated environments and shown that even within the general population, higher levels of interpersonal sensitivity and high anxiety are associated with increased tendency to interpret ambiguous behaviors such as looking, smiling and talking, as being more personally relevant and threatening (Freeman et al. 2003). In addition, service users with clinical levels of paranoia tend to interpret neutral social signs from the avatars abnormally and consistent with their paranoid beliefs (Freeman et al. 2005). Despite the limitations of extrapolating from the virtual to the real world, the combined evidence suggests that there may not be a direct link between the behavior of relatives and how this is perceived by the service user. This relationship may be partially mediated by the thoughts of the service user, and such thoughts may be a fruitful target of intervention in a CBT intervention based on an interpersonal framework.

### Evidence that Service User’s Behavior Impacts on Relative

There is evidence that certain service user behaviors and characteristics are more likely to elicit critical or hostile responses from relatives than others (link 9). Negative symptoms (Hooley et al. 1987; O’Brien et al. 2006; Weisman et al. 1998), substance misuse (Barrowclough et al. 2005) and violent behavior (Onwumere 2013) have all been identified as particularly likely to attract critical comments or hostility from relatives. More recent changes in behavior, and behaviors that persist seem to be viewed more negatively (MacMillan et al. 1986). Distress in relatives has also been linked to greater severity of symptoms, and younger age of onset (Addington et al. 2005; Barrowclough et al. 2014). However, what is more significant is the extensive evidence for the mediating role of relatives’ thoughts/appraisals in determining relatives’ emotional and behavioral responses to service user behavior (link 10).

### Relatives’ Responses are Partly Determined by Their Appraisals

Consistent with the cognitive model, there is now good evidence that relatives’ appraisal of the service user’s behavior is an important determinant of their emotional and behavioral response (link 10). First to explore this was Brewin et al. (1991) who found that carers rated as high EE on the basis of criticism or hostility were more likely to make controllable and personal attributions than over-involved or low EE carers (Brewin et al. 1991). Since then, there have been many studies exploring how relatives’ attributions impact on their responses (see Barrowclough and Hooley 2003 for a review). In summary, the attribution style studies suggest that relatives who behave in highly critical ways are more likely than those expressing low criticism to believe that service users are substantially in control of the negative events that relatives experience. They are also more likely to ascribe them greater personal responsibility for these negative events. Underlying personal responsibility attributions are judgements that the behavior of the service user is a result of factors that are internal and personal to that individual—but also could be controlled by them if they wished. Responsibility appraisals are even more apparent in relatives rated as hostile as well as critical. This helps explain why behaviors such as substance misuse, negative symptoms and violence are more likely to lead to critical or hostile responses in relatives. These behaviors are less obviously “symptoms” of an illness, appearing in the non-psychosis population and generally construed as under active control.

Most of the evidence for links between underlying beliefs and relatives’ behavioral responses (link 11) has come from coding of CFI transcripts. Hooley and Campbell (2002) used this methodology to demonstrate that, making more attributions of control is associated with behaving in more controlling ways, suggesting that the attributions may be driving behavioral responses which may in turn be linked to relapse in the service users. In contrast, relatives rated as high EOI, who tend to behave in ways that “buffer” the service user from the demands of life, tend to make very few attributions of responsibility to service user for any of their behaviors. This pattern has been described as “victim appraisals” in which the service user is seen as a victim of psychosis (Barrowclough and Hooley 2003). As a consequence, high EOI relatives often take a lot of responsibility for both the development of the psychosis, and the process of recovery. These associations were initially identified in a sample of relatives of people with chronic psychosis (Barrowclough et al. 1995; Hooley et al. 1987), but have recently been replicated in a recent onset group (Vasconcelos e Sa et al. 2013). Despite far less exploration of attributions associated with low EE, there is evidence to suggest that low EE relatives make what have been described as “survivor appraisals”. They tend to see the service user as less responsible for negative events than high EE critical relatives, but more responsible for positive events (Grice et al. 2009).

Relatives’ attributions also determine their emotional responses (link 13), in particular distress levels. Unsurprisingly, relatives who blame themselves for the mental health problems of their family member show higher levels of distress (Barrowclough et al. 1996; Boye et al. 2001; Fortune et al. 2005). This association has also been replicated in recent onset families, in which the most common self-blaming attribution was a perceived failure to recognise and respond to early signs of illness (Vasconcelos E Sa 2014).

Research exploring attributions underlying relatives’ responses has been immensely useful in guiding the development of our understanding of interpersonal dynamics in families of people with psychosis and in developing effective interventions which try to identify and modify attributions (e.g. Barrowclough and Tarrier 1992; Kuipers et al. 2002). This area of work is still developing, and recent advances include the wider exploration of beliefs about psychosis, beyond focussing on attributions about specific behaviors, and the insight that the interpersonal dynamic may be better understood as a function of the discrepancy between beliefs held by service users and relatives, rather than understanding just one perspective.

The Self-Regulation Model (Leventhal et al. 1984), applied to psychosis (Lobban et al. 2003) proposes that relatives develop working models of psychosis (as they would with any illness) which helps them to make sense of their experiences and guides their coping strategies. Specifically, they will hold beliefs along a number of dimensions including the identity of the illness, likely consequences, the controllability, the cause and the likely timeline. Barrowclough et al. (2001) found that the number of critical comments made by relatives was associated with a perceived greater frequency of symptoms, even when controlling for an objective measure of illness severity. The greater the criticism, the less sense there was of the illness being amenable to control/cure and the less able relatives felt to control the illness themselves. Finally, relatives rated as high EE perceived a more chronic timeline for the illness. This work suggests that a wider exploration of relatives’ beliefs that goes beyond attributions of control and responsibility may highlight other key beliefs that underlie distress in relatives, and or interpersonal difficulties with the service user, and which may provide fruitful targets for therapy. However, it is unlikely that understanding the relatives’ model of psychosis in isolation will provide the whole picture. EE reflects the quality of the relationship between the service user and relative from the relative’s perspective. Relationships are by definition between two or more people. Therefore, it is likely that the impact of beliefs held by the relative about the illness will depend upon how much they are in (dis)agreement with the beliefs held by the service user. Lobban et al. (2006) were the first to test this in psychosis and found that a comparison between models held by high and low EE relatives showed no significant differences between the groups—but when discrepancy scores were compared which showed the difference between the service user and relatives beliefs within each dyad, high EE dyads showed greater levels of discrepancy than was seen in low EE dyads, with the relatives tending to hold a more negative overall model of illness that then service user. Kuipers et al. (2007) used the same SRM framework with a larger sample of dyads. Although they found no direct link between illness beliefs and EE, they did show that the discrepant views were related to greater distress, depression and lower self-esteem in both service users and relatives. Taken together, these studies support the idea of more dynamic interpersonal application of the CBT framework in which the impact of beliefs is recognised as being dependent on the degree of discrepancy with those of significant others.

Consistent with the SRM, underlying attributions, appraisals, and illness beliefs are all important because they impact on the coping styles of the relatives, and on their emotional responses (links 13 and 11). As with much of the work in this area, there has been too little focus on understanding the underlying beliefs, appraisals, and working models of relatives who are able to manage psychosis without high levels of distress and who are able to successfully support the service user through the process of recovery. In addition to the identification of the “survivor appraisal style” characteristic of low EE relatives and described above, some interesting qualitative work (Treanor et al. 2013) has highlighted other key factors which may help us understand what we need to be working towards in supporting relatives. In a small study in which eight relatives rated as low EE on the CFI were interviewed in depth about their experiences of supporting a close relative with psychosis, the authors identified key themes underlying the relatives’ responses. The relatives shared an acceptance that they were unable to change what the service user was experiencing or doing—but an ongoing commitment to support them with managing these experiences. They demonstrated a deep emotional understanding of how the service user was feeling, and had complex working models of the cause and maintenance of the problems. Coping styles focussed around humour, distraction and time out, and downward social comparison—recognising that things could be (and often are) a lot worse for others. Characteristic of the relatives interviewed was the presence of realistic optimism for the future, characterised by an acceptance of a change in life course for the service user rather than perceiving a failure to achieve previously identified goals. This preliminary work highlights how much more we can learn from in-depth interviews with relatives who have already nurtured the relationships we aspire to achieve through clinical interventions.

Finally, the SRM (and other cognitive models) would suggest that behavior is not determined by cognitive representations alone—but also directly by emotional representations, and there is some evidence to support this assertion in relation to relatives’ responses to psychosis (link 12). Anxiety, fear and grief have all been explored in this context. Relatives’ coping strategies and behavioral responses to the service user may reflect their attempts to manage their emotional responses to psychosis and allowing relatives to express and work through these emotions may facilitate behavior change. For example, Greenley (1986) found that high EE was associated with relatives being more fearful and anxious and suggested that their controlling behavior was a way of trying to manage these emotions. Patterson et al. (2005), showed strong links between high EOI and loss, and suggested that use of buffering behaviors were attempts by the relative to deal with loss, and that over time this loss would either reduce, with an associated switch towards low EE and more supportive behavior, or remain and this could lead to critical and controlling behavior in an attempt to change the situation and get the service user to return to pre-morbid functioning. This has interesting implications for working therapeutically with relatives to facilitate the grieving process, and also highlights the dynamic nature of relatives’ responses and factors that may influence change over time.

## Interpersonal CBT Framework: What Does It Add?

In this paper we have presented a framework that extends the CBT model to include the role of relatives’ behavior in the development and maintenance of psychosis. We have used this framework to make the case for greater involvement of relatives in psychosocial interventions, highlighting the increased opportunities to effect change, and improve outcome for both service users and relatives. There are other frameworks which use the CBT model to understand cognitive interpersonal processes underlying relapse in psychosis (e.g. Burbach 2013; Gumley et al. 1999). In fact, most of the cognitive models of psychosis highlight the important role of friends and relatives in the development and maintenance of psychosis (Bentall et al. 2001; Freeman et al. 2002; Garety et al. 2001; Morrison 2001; Steel et al. 2005). However, these models tend to focus in detail on changes in the cognition, emotion and behavior of the service user and see the relatives’ behavior as an external variable triggering this, rather than using the cognitive framework to conceptualise how the relative and service user interact in a dynamic way, as we have done here. In contrast, the cognitive model of care-giving proposed by Kuipers et al. (2010) focuses on the changes in cognition, emotion and behavior of the relative, and how these also impact on relatives’ outcomes. This model suggests that relatives’ behavioral responses to the service user are determined by both cognitive and behavioral changes in the carer which are in turn determined by their appraisal of the illness, the specific behavior of the service user and their relationship with services. The model is very useful in understanding the variation in how relatives seek help and in determining the kind of support they are most likely to benefit from. It works as a formulation for successful as well as problematic relationships. A key strength of the model is that builds on a cognitive model which many clinicians are already familiar with. However, the model covers only the relatives’ response. We believe that the Interpersonal CBT Framework presented here builds on both the cognitive model of care-giving, and on cognitive models of service user experiences in psychosis by attempting to understand the nature of the interaction and how the behavior of each person can impact on the cognition, emotion and behavior of the other.

## Limitations and Areas for Further Research

We hope that the Interpersonal CBT Framework will be successful in elaborating existing CBT models of psychosis to increase understanding of the role of the social environment and to offer clinicians a framework for greater involvement of relatives in CBT interventions, leading to a more systemic approach to psychosis. However, there a number of limitations with the current framework which can only be addressed through further research.

Firstly, although there is extensive research supporting some parts of the mechanism proposed, evidence for other aspects is limited. The framework hypothesises links which require formal testing. Specifically, the framework proposes that changes in the behavior, emotion, or appraisals of either the relative or the service user, could effect change in the dynamic interpersonal system. These hypotheses could be tested directly in intervention studies in which the mechanism of change is evaluated, as well as the outcomes. A comparison of interventions focussing on behavioral, cognitive, or affective change would further identify which elements are most amenable to change. The hypothesised direct link between behaviour of the relative and the emotion and arousal response of the service user (link 7) and vice versa (link 14), in particular requires further testing. Unlike traditional CBT models (such as Beck 1979), in which appraisals mediate the link between events and emotion (link 2), or behaviour (link 4), there is growing evidence of multiple routes to emotion and behaviour change, not all of which are mediated by appraisals. The exact nature of the relationship between emotion and cognition remains a hotly debated topic, with as yet no definitive answer (Eder et al. 2007). Hence, in our Interpersonal CBT framework we retain a direct link between the behaviour of the relative and the emotion and arousal of the service user (link 7) and vice versa (link 14) in order to acknowledge possible causal mechanisms which are not mediated by appraisals.

Secondly, the evidence we use to support the Interpersonal CBT Framework, draws predominantly on research aimed at understanding interpersonal processes that have been associated with negative outcomes, such as high EE, distress and relapse. We have highlighted throughout the need to understand interpersonal processes associated with positive outcomes, but to date this literature is limited. Relationships that have a positive impact on mental health are characterised not just (and possibly not even) by the absence of processes underlying interactions detrimental to mental health, but by the presence of additional features which we need to better understand in order to focus our interventions around building these features, rather than just on overcoming problematic dynamics. For example, the extensive research into relatives’ thoughts, feelings and behaviors underlying high EE responses, needs to be extended to more fully understand those same processes underlying low EE responses, and warm supportive relationships more broadly, shifting the focus from “what are relatives doing wrong?” to “how do some relatives manage so well?” Further, understanding the processes by which relatives come to form such varying models of understanding and different coping responses is crucial if we are to improve early interventions that can facilitate supportive social environments so important in determining recovery.

Thirdly, much of the evidence cited to support the Interpersonal CBT Framework is derived from research methods involving retrospective self-report and laboratory based observation of behavior. Such methods limit the strength of conclusions that can be drawn about the causal nature of relationships hypothesised in the model and their ecological validity. Stronger support would come from research using techniques which can capture the interpersonal dynamics as they occur in real time and in real world settings. For example, measuring the thoughts, feelings and behaviors of service users and relatives, as they occur in their everyday lives, and in relation to their interactions with one another, would greatly enhance our understanding of which variables are driving which. Methodologies such as Experience Sampling Methods (ESM) provide an opportunity to do this by taking snapshots of variables of interest at random times throughout the day. This data, including the relationships between variables can be tested using longitudinal models which explore which variables precede others, strengthening the case for a causal relationship. We can further test the causal nature of these relationships using intervention studies that aim to change key thoughts, feelings or behaviors that seem to be driving problematic interpersonal dynamics, and strengthen those associated with virtuous cycles.

Finally, the framework outlined in Fig. 1 represents the interpersonal dynamic between a service user and one relative. The focus is on an in-depth understanding of one close significant relationship, which is likely to have a strong impact on wellbeing, and which may offer an opportunity for engagement in therapy. Despite the evidence that people with psychosis have reduced social networks, the impact of the social environment on their mental wellbeing is likely to extend beyond one person. The framework can be developed in a number of different ways in order to capture this complexity, and this is demonstrated using the case example below.

## Clinical Implications and Case Example

The Interpersonal CBT Framework has several key features which make it flexible enough to be used across the array of family structures, and presenting problems seen in real world clinical services, and to inform interventions at different levels of intensity:

1. The framework builds on existing CBT knowledge and skills already widely available in clinical practice. Staff do not require training and supervision in a whole new paradigm, but can use the framework to involve relatives in therapy as a natural extension of current clinical practice.
2. The framework can be adapted for any kind of relationship. Although much of the evidence draws on data collected from immediate family members, increasing recognition of the importance of broader social networks (Priebe et al. 2013) can also be accommodated within this framework.
3. The framework is explicitly designed to accommodate positive formulations in which service users and relatives understand the way in which their behaviours can positively impact on their own thoughts and feelings, and those of other people. This approach is consistent with the increasing focus on recovery in clinical services, in both the US (Department of Health and Human Services 2003) and the UK (NICE 2014). An example of a positive formulation is described in the case example below.
4. The framework offers a guide to interventions, but is not prescriptive in its approach. It is used to inform the formulation process, but the exact nature of the intervention will also depend on the needs and wishes of the service user and relatives(s), training of the clinician, and resources available.

### Case Example

This case example is based on clinical experience and designed to demonstrate how the framework can be used to inform clinical practice. Any similarity to any real person is entirely coincidental.

### Case Example: Parental Relationship, Demonstrating Use of Vicious and Virtuous Cycles Using the Interpersonal CBT Framework

**Fig. 2:**
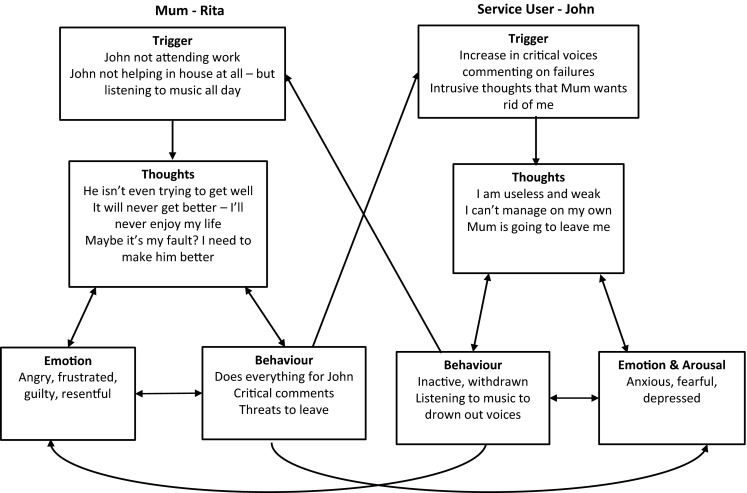
Vicious cycle using interpersonal CBT framework

This case example demonstrates how a simple individual CBT formulation of a young man (John) can be extended using the Interpersonal CBT Framework to understand how his relationship with his mother (Rita) plays a role in the maintenance of his symptoms (Fig. 2) and the process of his recovery (Figure 3—online resource). The framework helps to identify the opportunities for clinical interventions that can achieve the shift from the vicious cycle in which psychosis is maintained, into a virtuous one in which John’s mental health begins to improve, and mother’s distress is reduced.

### Background

John is a 35 year old man who lives with his mother, Rita. He has a part time placement at a local organic gardening centre which supports people who have mental health difficulties to get back into work by offering structured activity and support. John had a fairly happy childhood and did well academically. John first experienced mental health problems aged 17, during his first job as a salesman for a mobile phone company. He didn’t enjoy the job, found the targets stressful, and felt bullied by his line manager who gave him a lot of negative feedback, despite his attempts to work hard and achieve the targets set. This made him feel depressed and he started to miss work, making it even more difficult to hit his targets. John got more and more stressed as he felt stuck in the job because he wanted to help his mother financially by paying for his rent and food at home. Eventually John stopped going to work altogether. He became very low in mood and stayed in bed all day.

John’s mother, Rita, was both frustrated with him for not getting up, but also worried about his mood. At times she would get angry with him and shout at him to get out of bed and get on with his life. At other times she felt very sorry for him tried to look after him by bringing him food, and DVDs to watch. Rita became very anxious when she heard John talking to himself and she suspected John was hearing voices. Eventually Rita contacted the GP and John was assessed at home. Following a few weeks of visits by the mental health team, John was diagnosed at the age of 18 as having a psychotic episode, and prescribed antipsychotic medication which he agreed to take. Since this time John has had periods of time when he feels well and is able to enjoy life and works as a part time labourer for a friend’s business. However, he has also experienced several episodes in which his mood has become very low and he has started to hear voices again. These are generally triggered by an argument, disagreement or negative feedback from someone. These episodes have been managed by a combination of medication and talking to his Care Coordinator with whom he has a good relationship. On the whole, John also has a good relationship with his mum. However she is finding John’s episodes increasingly difficult to manage. The Care Coordinator noticed that the levels of arguments between John and his mum greatly increase during John’s episodes of psychosis, and that these arguments were getting more heated over time. She felt this was having a negative impact on both John and Rita and shared this thought with them. They both agreed and were keen for some help to try and manage things differently.

### Formulation

Figure 2 provides a formulation developed by John’s Care Coordinator which was based on the Interpersonal CBT Framework. This was informed initially by her discussions with John and his mum, and her observations of the family dynamic over a period of 6 months of visiting the family. Once the family had expressed a wish to work on this issue, she met with John and Rita together, and with each individually, and asked them in greater detail about their perspectives on the problem. She developed the formulation jointly with them by getting them to talk through recent difficult arguments and concerns, and drawing out their thoughts, feelings and behaviours during these times.

This diagram highlights the interpersonal dynamic between John and Rita and shows how this is maintaining negative emotions for both of them. Rita feels angry and frustrated with John when he withdraws from work and friends as she can see that this makes him become depressed and the voices worse. She can’t understand why he doesn’t do things to make himself feel better, and worries she will always have to look after him, which is exhausting as Rita also works long hours in order to pay their living costs. At the same time, Rita feels very sorry for John, and wonders if she is in fact to blame for his problems, as she is the person who brought him up. This makes her feel very guilty and she wants to make up for what she sees as her own failings as a parent and so she tries to care for him by doing all the household tasks which leaves her feeling exhausted. John is very sensitive to his mother’s frustration. When she is cross with him, it makes him feel more anxious and depressed. He wants to feel more independent but at the same time is terrified that she will get fed up and will leave him, and so he continues to rely on her, partly as a strategy to ensure she stays.

### Intervention

The Care Coordinator, John and Rita used the framework to identify things that could be changed in order to shift the interpersonal cycle from a vicious cycle which maintains the problems, to a virtuous cycle which facilitates a positive and supportive relationship. The intervention involved the following:

1. Behavioural strategies
  - John and Rita planned to spend more time together doing things they both enjoy to strengthen the positive aspects of their relationship e.g. both Rita and John really enjoy food, so this started with Rita teaching John how to cook some of his favourite dishes.
  - Rita agreed to support John to gradually take on more tasks around the house to build his confidence in own skills e.g. cooking together resulted in John cooking dinner one night each week. Rita was very impressed and able to share this with John which made him feel great.
2. Cognitive strategies
  - As John reviewed the skills he did have around the house, and the new skills he was developing, he was able to challenge his thoughts that he was useless, and that he would not be able to manage on his own.
  - John and Rita were encouraged to discuss their thoughts about the future. Rita was surprised to learn that John wanted to eventually to live alone, but was anxious about losing contact with his mum as he felt she was the only person who understood him. Rita was able to reassure him that if he ever did feel confident enough to live on his own, she would still be nearby to support him. This gave John the confidence to develop his independence without fearing the loss of his relationship with his mother.
  - The Care Coordinator spent some time individually with Rita to explore her thoughts about being to blame for John’s mental health problems. Together they reviewed the evidence available in written literature for the causes of mental health problems, and considered the relevant events in John’s life. Rita’s causal model of John’s problems changed from one in which her parenting played a major role, to one in which she recognised John as having always been a very sensitive person who finds interpersonal criticism particularly upsetting. This was highly exacerbated by the trauma of his first job experience and the stress of this caused John to become depressed and hear voices. This new model meant that Rita felt less to blame, and was also more attuned to how her own interactions with John might impact on his mental wellbeing. Understanding his increased sensitivity to criticism, resulted in her trying to convey any dissatisfaction with John’s behaviour in a more constructive way, and being more vocal in sharing her positive feelings towards any progress he was making.
    The resulting virtuous cycle (Figure 3—online resource) was developed with Rita and John and used to continue to guide progress.

### Extending the Framework to Work with Three People

The case example of John and Rita describes how the interpersonal CBT framework can be used to inform interventions with a parent and child dyad. We chose this scenario as it’s possibly the most common presentation in mental health services. However, the framework can also be used when working with more than two relatives. Below we extend the formulation for John and Rita, to include the role that Johns’ stepdad Ron plays.

Ron would like to see John take more responsibility for his life and rely less on Rita, because he worries about how exhausted Rita seems a lot of the time. He sometimes tries to have a word with John about this, but this makes John feel more criticised and also worried that Ron is trying to persuade Rita to move in with him, which would leave John on his own. This fear is reinforced by Ron’s attempts to take Rita away on holidays. Ron also tries to talk to Rita about how she parents John as he thinks she is too soft with him. This reinforces Rita’s belief that John’s problems have occurred as a result of her parenting throughout his life.

This formulation highlights the need to involve Ron in the intervention otherwise his behaviour could undermine the changes John and Rita are trying to make. However, Ron works fulltime and Rita felt it would be too much to ask him to get involved in the therapy sessions with John’s Care Coordinator on a regular basis. They agreed to invite Ron to two key sessions. In the first, they shared the formulation in Fig. 2 with Ron and elicited his views on what was happening. This resulted in additions being made to the formulation to account for Ron’s thoughts, feelings and behaviours and the influence these have on Rita and John (see Figure 4—online resource). Once Rita and John had agreed a plan to move forward, they decided to invite Ron to a further session in which they shared their plan with him. Ron was then able to understand the importance of Rita and John spending more positive time together and of Rita helping John to learn new skills around the house. He was also able to understand John’s increased sensitivity to criticism and agreed to follow Rita’s example in trying to notice small gains in John’s attempts to become more independent and focussed on making lots of positive comments to support these improvements. Ron had not understood John’s fears of losing contact with his mother and was able to reassure John that this was not his intention.

The Interpersonal CBT Framework can in theory be adapted to work with a range of different interpersonal structures. However, whilst it is possible to imagine how the framework can be used to understand the interactions between more than three people, it becomes increasingly more difficult to represent in 2D paper format.

The framework can be used to work with a variety of relationships (friends, partners, siblings, children), and to facilitate interventions at a range of different levels depending on need including: a supported self-management approach providing information and support for the relatives (e.g. Lobban et al. 2013a); involving relatives in a joint CBT focussed intervention to facilitate development of a shared working model and support implementation of specific cognitive and behavioural strategies; structured family focussed therapy (e.g. Barrowclough and Tarrier 1992; Kuipers et al. 2002). Unfortunately, due to constraints of space we cannot provide detailed case examples of each of these scenarios.

## Conclusion

The Interpersonal CBT Framework offers a theoretical basis on which a wide range of interventions, targeting key specific elements, can be developed, and which makes specific testable predictions about outcomes. It builds on the individual CBT framework which is already familiar to many clinicians working in mental health services, and therefore is more likely to be adopted without the barrier of extensive and expensive additional training. The framework highlights the importance of the interpersonal environment in the process of recovery, but does not suggest that psychosis is caused by the behaviour of relatives, or that relatives’ responses to psychosis are pathological. In fact, the framework presents interpersonal responses to psychosis as entirely predictable responses to an often very challenging experience. We anticipate that the framework will facilitate increased engagement of relatives in routine clinical services where this is appropriate.

There are limitations to the clinical applicability of the framework. Firstly, as it explicitly builds on existing CBT knowledge and skills, it is of limited use to clinicians who have not had this training. However, given that individual CBT models have been developed for most common mental health problems and extensive national and international infrastructures exist to support training, it is likely that the majority of mental health clinicians have at least a basic understanding of the CBT model.

Secondly, we acknowledge that in some circumstances involving relatives this may not be appropriate, for example in cases of deliberate ongoing or potential abuse within the relationship. In our experience, this is rare among relatives seeking support in routine clinical services.

Thirdly, according to prominent implementation theories, important factors influencing the likelihood of a new clinical intervention being taken up successfully within existing services are not in place. For example, the Promoting Action on Research Implementation in Health Services framework (PARIHS) proposes that successful implementation (SI) is a function of the nature of the evidence available (E), the context (C) in which the evidence is being introduced, and the way the process is facilitated (F), where SI = f(E, C, F) (Kitson et al. 2008; Rycroft-Malone et al. 2002). In its favour, the Interpersonal CBT framework has been developed specifically to build on existing frameworks familiar to clinicians, and is flexible in being able to guide interventions across a range of levels of intervention, and in this sense is complimentary to the existing context (C) in which it is being implemented. However, the evidence base (E) and facilitation process (F) are more limited. Although there is extensive evidence to support the structure of the framework (outlined above), the effectiveness of interventions based on this framework have not been formally tested. We have used the framework to guide our own clinical interventions in routine clinical practice and have shared the framework with other clinicians in workshops at national and international conferences: extensive positive feedback from which has inspired us to write this paper. We hope that existing infrastructures to support training and supervision for CBT can be elaborated to incorporate the interpersonal CBT framework with the aim of enhancing the confidence and skills of clinicians in working with relatives as a natural progression of their current CBT practice.

## Electronic supplementary material

Supplementary material 1 (PPTX 63 kb)

Supplementary material 2 (PPTX 72 kb)
