# Supplementary material for: An Interpersonal CBT Framework for Involving Relatives in Interventions for Psychosis: Evidence Base and Clinical Implications
Source: Cognit Ther Res. 2015 Dec 11;40:198–215. doi: 10.1007/s10608-015-9731-3 (PMC4792366; doi:10.1007/s10608-015-9731-3)
Supplement: Supplementary file 1 — Supplementary material 1 (PPTX 63 kb) [file 10608_2015_9731_MOESM1_ESM.pptx]

## Slide 1
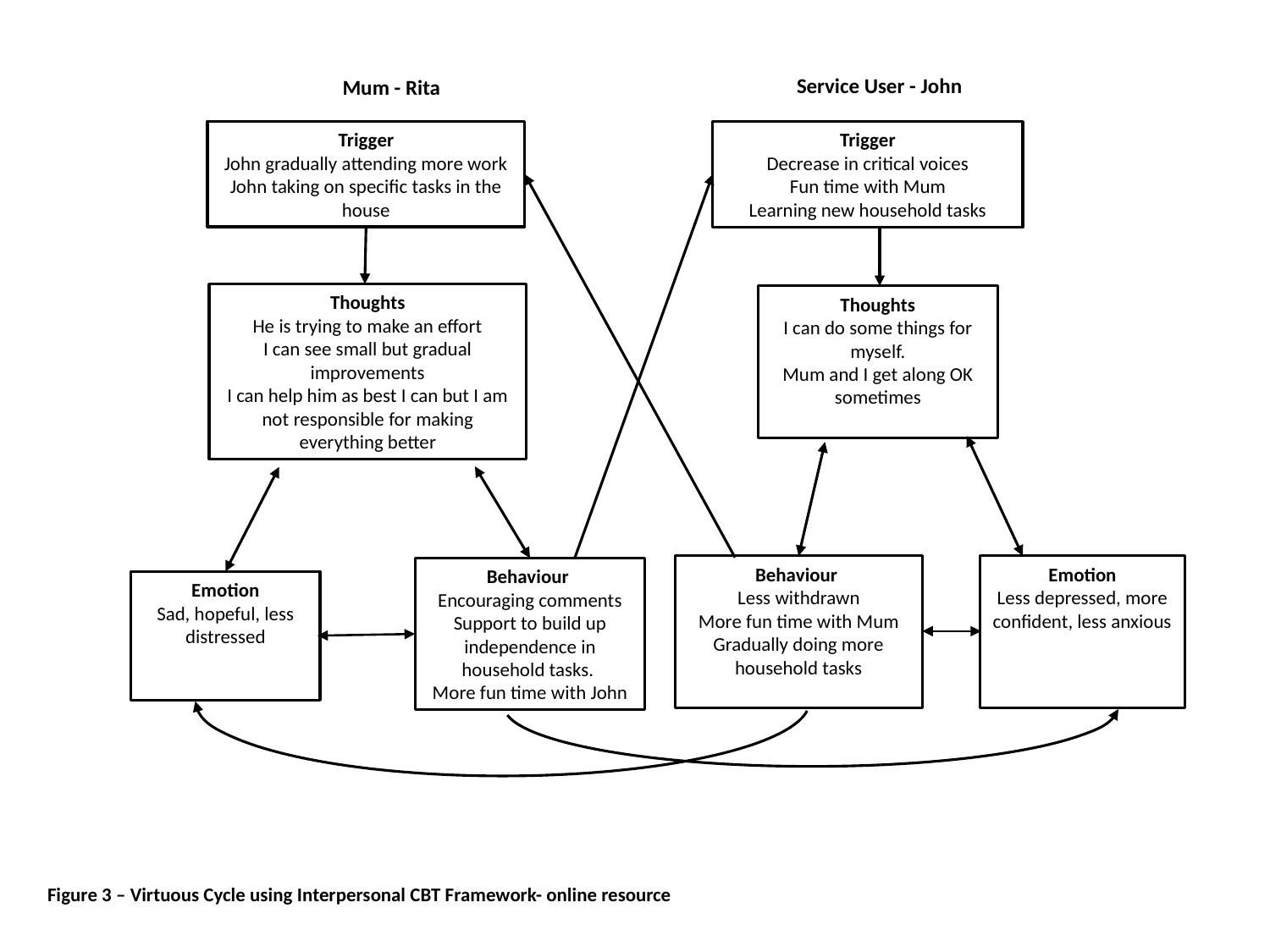

Service User - John
Trigger
Decrease in critical voices
Fun time with Mum
Learning new household tasks
Thoughts
I can do some things for myself.
Mum and I get along OK sometimes
Behaviour
Less withdrawn
More fun time with Mum
Gradually doing more household tasks
Emotion
Less depressed, more confident, less anxious
Mum - Rita
Trigger
John gradually attending more work
John taking on specific tasks in the house
Thoughts
He is trying to make an effort
I can see small but gradual improvements
I can help him as best I can but I am not responsible for making everything better
Behaviour
Encouraging comments Support to build up independence in household tasks.
More fun time with John
Emotion
Sad, hopeful, less distressed
Figure 3 – Virtuous Cycle using Interpersonal CBT Framework- online resource
