# Supplementary material for: An Interpersonal CBT Framework for Involving Relatives in Interventions for Psychosis: Evidence Base and Clinical Implications
Source: Cognit Ther Res. 2015 Dec 11;40:198–215. doi: 10.1007/s10608-015-9731-3 (PMC4792366; doi:10.1007/s10608-015-9731-3)
Supplement: Supplementary file 2 — Supplementary material 2 (PPTX 72 kb) [file 10608_2015_9731_MOESM2_ESM.pptx]

## Slide 1
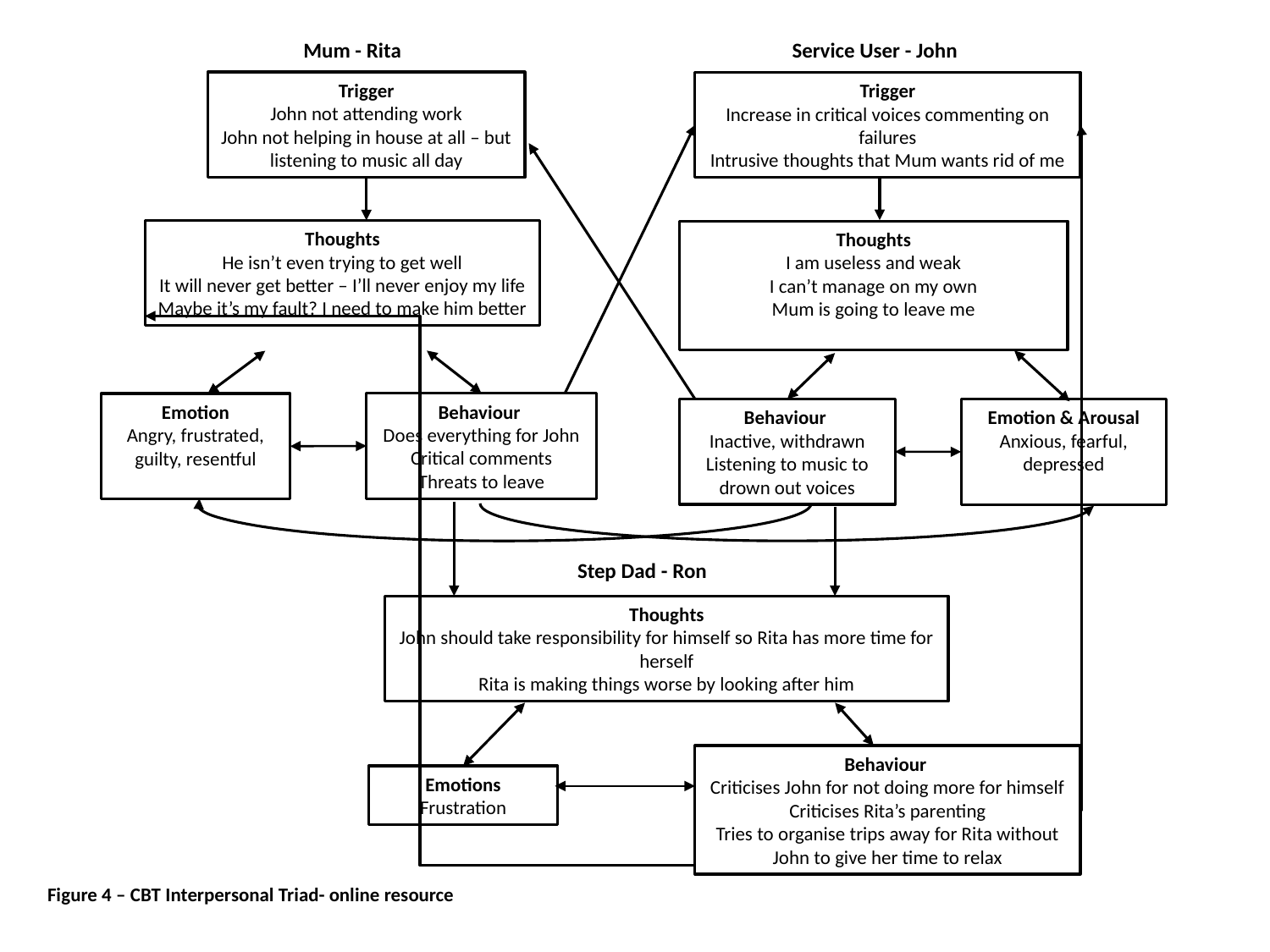

Mum - Rita
Trigger
John not attending work
John not helping in house at all – but listening to music all day
Thoughts
He isn’t even trying to get well
It will never get better – I’ll never enjoy my life
Maybe it’s my fault? I need to make him better
Behaviour
Does everything for John
Critical comments
Threats to leave
Emotion
Angry, frustrated, guilty, resentful
Service User - John
Trigger
Increase in critical voices commenting on failures
Intrusive thoughts that Mum wants rid of me
Thoughts
I am useless and weak
I can’t manage on my own
Mum is going to leave me
Behaviour
Inactive, withdrawn
Listening to music to drown out voices
Emotion & Arousal
Anxious, fearful, depressed
Step Dad - Ron
Thoughts
John should take responsibility for himself so Rita has more time for herself
Rita is making things worse by looking after him
Behaviour
Criticises John for not doing more for himself
Criticises Rita’s parenting
Tries to organise trips away for Rita without John to give her time to relax
Emotions
Frustration
Figure 4 – CBT Interpersonal Triad- online resource
